# CD45 and Basigin (CD147) Are Functional Ligands for Galectin-8 on Human Leukocytes

**DOI:** 10.3390/biom15091243

**Published:** 2025-08-27

**Authors:** Jean-Philippe F. Gourdine, Porfirio Nava, Alexander J. Noll, Duc M. Duong, Nicholas T. Seyfried, Richard D. Cummings

**Affiliations:** 1 Chemistry Department, Lewis & Clark College, Portland, OR 97219, USA; jgourdine@lclark.edu; 2 Biochemistry & Molecular Biology Program, Lewis & Clark College, Portland, OR 97219, USA; 3 Department of Physiology, Biophysics, and Neurosciences, Center for Research and Advanced Studies of the National Polytechnic Institute (CINVESTAV-IPN), Mexico City 07360, Mexico; 4 The Emmes Company, LLC, Rockville, MD 20850, USA; 5 Department of Biochemistry, Emory University School of Medicine, Atlanta, GA 30329, USA; 6 Department of Surgery, Beth Israel Deaconess Medical Center, Harvard Medical School, Boston, MA 02215, USA

**Keywords:** CD45, basigin, galectin-8, leukocytes, glycan ligands

## Abstract

The interactions of leukocyte glycoproteins with adhesion and signaling molecules through glycan recognition are not well understood. We previously demonstrated that galectin-8, a tandem-repeat lectin with N- and C-terminal carbohydrate binding domains which is highly expressed in endothelial and epithelial cells, can bind to activated neutrophils to induce surface exposure of phosphatidylserine (PS) without DNA fragmentation or apoptosis, in a process termed *preaparesis*. However, the receptors for Gal-8 on leukocytes have not been identified. Here we report our results using both proteomics and affinity chromatography with both full-length Gal-8 and the separate Gal-8 C-terminal and N-terminal domains to identify glycoprotein ligands in HL-60 cells for Gal-8. Two of the major ligands for Gal-8 are CD45RA and CD45RC (Protein Tyrosine Phosphatase, PTP) and basigin (CD147). Both CD45 and basigin are integral membrane glycoproteins that carry poly-N-acetyllactosamine modifications on N- and/or O-glycans, required for Gal-8 binding. Inhibition of the phosphatase activity of CD45 reduced Gal-8-induced PS exposure, indicating a possible role of CD45 in Gal-8 signaling of *preaparesis* in human leukocytes. These results demonstrate unique glycoprotein recognition by Gal-8 involved in cell recognition and signaling.

## 1. Introduction

The molecular interactions of leukocytes with other cells in homeostasis and in inflammation encompass a wide range of adhesive interactions and signaling events involving many families of glycan-binding proteins, including selectins, Siglecs, and galectins [1,2]. The latter have a highly conserved carbohydrate recognition domain (CRD) acting through key amino acids such as H61, N63, and R65 in the Gal-8 C terminal domain [3], and are involved in many processes including innate immunity [4], T-cell signaling [5], and leukocyte turnover [6,7,8,9,10]. Certain galectins, including Gal-1, Gal-2, Gal-4, and Gal-8, have the ability to induce non-apoptotic phosphatidylserine (PS) exposure on the external leaflet of the plasma membrane, termed *preaparesis*, on activated neutrophils and HL-60 cells [8,10,11]. HL-60 cells are human promyelocytic leukemia cells commonly used as a model system for leukocyte biology [12]. The galectin-induced non-apoptotic PS exposure represents a new paradigm of leukocyte clearance without the spread of detrimental biomolecules released by dying cells, which could perpetuate inflammation [13]. Whereas galectin receptors on T–cells are documented to an extent [14], little is known about receptors for galectins on neutrophils [1,15]. The precise receptors for the galectins on neutrophils are poorly understood.

The tandem repeat Gal-8 induces *preaparesis* on HL-60 cells through its C-terminal domain, which binds to blood group antigen epitopes and poly-N-acetyllactosamine (polyLacNAc) glycans [8]. As Gal-8 is expressed in endothelial cells, smooth muscle cells, and many other cell types [16,17,18], upon its secretion it could interact with neutrophils during their extravasation. While many binding partners for various galectins have been identified, the glycoprotein ligands on leukocytes and particularly neutrophils for Gal-8 have not been comprehensively studied [19]. It is important to study Gal-8 receptors on neutrophils as they are likely involved in non-apoptotic PS exposure, leading to phagocytic removal of living cells without inflammation [8]. Since their discovery, HL-60 cells have been a cell line of choice to model neutrophils in different interaction studies [7,8,20,21,22]. Therefore, we investigated human HL-60 cells, a promyelocytic cell line, to identify ligands for Gal-8 using Gal-8 affinity chromatography followed by proteomic analyses. Here we identified several glycoprotein ligands for Gal-8, and among the most prominent are CD45, a tyrosine phosphatase, and basigin, which is also known as CD147. CD45, a polyLacNAc-containing glycoprotein expressed by all nucleated hematopoietic cells [23], has been shown to be involved in regulation of PS exposure in B-lymphocytes [24]. Basigin is an extracellular matrix metalloprotease inducer (EMMPRIN), OK Blood group antigen, and leukocyte activation antigen M6. Whereas basigin interactions with Gal-3 have recently been reported for human retinal pigment epithelial cells [25], we present here the first observation of basigin/Gal-8 interactions on leukocytes. We further characterized this Gal-8–basigin interaction and demonstrated that it requires polyLacNAc on N-glycans. As noted in a recent review by Zhang et al. [2], even if all galectins bind to polyLacNAc, there are subtle differences in the binding to glycoproteins bearing polyLacNAc epitopes. For instance, the Gal-8 N- and C-terminal domains both bind to polyLacNAc but only Gal-8C is capable of signaling PS exposure. The results demonstrate the unique properties of leukocyte cell surface glycoproteins and functions of glycans through recognition by Gal-8, and the potential to regulate leukocyte signaling.

## 2. Materials and Methods

### 2.1. Membrane Preparation

HL-60 and Chinese hamster ovary (CHO) Lec8 cell lines, obtained from American Type Culture Collection (ATCC), were maintained at 37 °C and 5% CO_2_ in RPMI 1640 medium supplemented with 10% fetal bovine serum, 2 mM glutamine, 100 milliunits/mL penicillin, and 100 µg/mL streptomycin. Cells (10^9^ to 10^10^) were suspended in phosphate buffer saline (PBS) pH 7.4 with protease inhibitor cocktail (Roche) and lysed using the freeze-and-thaw method with liquid nitrogen and ice for two cycles. Cell disruption was verified between each cycle by microscopy. Cell debris was pelleted by centrifugation (200× *g* at 4 °C for 10 min). The supernatant was centrifuged at 100,000× *g* at 4 °C for 30 min in a Beckman Coulter L90K ultracentrifuge. Pellets were solubilized in PBS-1.5% Triton X-100 (PBS-TX) and incubated on ice for 30 min. Membrane preparations were centrifuged at 200× *g* at 4 °C for 10 min to remove the insoluble material. Protein concentration was estimated using bicinchoninic acid (BCA) assay according to the manufacturer’s protocol (Thermo Fisher Scientific, Rockford, IL, USA).

### 2.2. Coupling of Gal-8 to Affi-Gel-10 Beads

Gal-8, Gal-8-C terminal domain (Gal-8C) and Gal-8-N terminal domain (Gal-8N) were expressed in *Escherichia coli*, purified on a lactosyl-sepharose column, and then stored in PBS-14 mM β-mercaptoethanol (BME) with 100 mM lactose as described previously and as needed for galectins, due to the free cysteine residues that can create disulfide bonds and subsequent aggregation and inactivation [8,26]. Gal-8 and its two domains (1–10 mg) were separately coupled to 1 mL Affi-Gel-10 beads (Bio-Rad, Hercules, CA, USA) according to the manufacturer’s protocol using the cold aqueous coupling method, with minor changes. The coupling was carried out in the presence of 100 mM lactose, to prevent the coupling of Gal-8 to Affi-Gel through the amino acid residues in the CRD essential for the glycan binding. Unoccupied (free) sites on Affi-Gel were quenched with 1 mL of 1 M glycine under rotation overnight at 4 °C. An aliquot of each Gal-8 solution (before and after coupling) was saved for sodium dodecyl sulphate–polyacrylamide gel electrophoresis (SDS-PAGE) and protein assay with BCA method. As a control, 1 M glycine was coupled to Affi-Gel-10 beads under the same conditions to serve as control glycine beads.

### 2.3. Gal-8 Affinity Chromatography of HL-60 Cell Membrane Preparation or Intact Cells

HL-60 membrane preparation (0.5 mL, 250 µg/mL protein) was incubated either with 1 mL of Gal-8-beads (0.5, 1, 2, 5, and 10 mg/mL) or glycine-beads (1 M) in a 10 mL polypropylene pipette plugged with glass wool (amorphous silicate). Fractions were collected as follows: flow through, wash (2 fractions of 5 mL each eluted by PBS-TX), elution of unbound material (2 fractions of 1 mL each eluted by 100 mM sucrose in PBS-TX), and specific elution by 100 mM lactose in PBS-TX (2 to 3 fractions of 1 mL each). For some experiments, flow through, wash, and sucrose fractions were pooled as unbound fractions (U) and lactose fractions were collected as bound fractions (B). Proteins in U and B fractions were precipitated with 10% trichloroacetic acid (TCA). PolyLacNAc-rich mouse glycoprotein laminin (Invitrogen) was used as a positive control (200 micrograms) and the membrane preparation from CHO-Lec8 cells (lacking polyLacNAc) was used as a negative control for the affinity chromatography on Gal-8 beads. HL-60 cell membrane preparation was used with glycine-beads for an additional negative control. For Gal-8 affinity chromatography of intact cells, HL-60 cells were incubated with 1 mL of Gal-8-beads at 4 °C for 1 h under rotation. TX was added to 1.5% final concentration to the mixture of Gal-8 beads interacting with HL-60 cell and rotated for 30 min at 4 °C. The mixture was passed over a glass wool column to remove impurities. Affinity chromatography was performed as described above. While *Datura stramonium* agglutinin (DSA) or *Lycopersicon esculentum* lectin (LEL) bind to polyLacNAc, they do not induce non-apoptotic PS exposure, contrary to Gal-8. Even though both Gal-8 domains bind to polyLacNAc, only the Gal-8C domain can induce non-apoptotic PS exposure. These differences between ligands could only be deciphered using Gal-8 columns.

### 2.4. Gal-8 Affinity Chromatography of HL-60 Cells After Cell Surface Biotinylation

HL-60 cells (25 × 10^6^ cells) were biotinylated using membrane-impermeable EZ-link sulfo-NHS-biotin according to the manufacturer’s protocol (Thermo Fisher Scientific, Rockford, IL, USA), then resuspended in 8 mL PBS. Four milliliters of biotinylated HL-60 cells were incubated either with Gal-8-beads or glycine-beads for 1 h at 4 °C under rotation. After adding TX to 1.5% final concentration, affinity chromatography was performed as above. After SDS-PAGE and transferring the blot with Transblot (Bio-Rad, Hercules, CA, USA), biotinylated cell surface proteins were detected by Streptavidin-horseradish peroxidase (Strep-HRP, Vector Labs, Newark, CA, USA) at a 1:30,000 dilution in Tris buffer saline (TBS) containing 0.1% Tween-5% bovine serum albumin (TBST-BSA) after blocking overnight at 4 °C or 1 h at room temperature (RT) in TBST-BSA. After three wash steps, membranes were incubated in 5 mL of Supersignal Pierce West pico enhanced chemiluminescent substrate (ECL, Thermo Fisher Scientific (previously Pierce), Rockford, IL, USA) for 1 min, then exposed for 5 min with Hyblot CL films (Denville Scientific, Holliston, MA, USA).

### 2.5. Glyco-Enzymatic Treatment of HL-60 Cells

To ensure that Gal-8 binding is carbohydrate dependent, HL-60 cells or HL-60 membranes were mock-treated with buffers alone, or PNGase F or endo-β-galactosidase (from *E. freundii*) following the manufacturer’s protocol (NEB, Ipswich, MA, USA), then used in Gal-8 affinity chromatography columns as described above.

### 2.6. Gal-8 Binding Assay

Two hundred microliters of each fraction eluted from the Gal-8-Affi-Gel-10 beads (200 µL each) was coated on a black polystyrene plate (Costar) by incubation at 37 °C for 1 h. Wells were blocked with 200 µL of PBS-5% BSA at 37 °C for 1 h, then incubated with 50 µg/mL biotinylated-Gal-8 at 37 °C for 1 h. The biotinylated-Gal-8 was prepared as previously described [6]. Streptavidin-Alexa 488 (Strep-Alexa 488, 100 µL of 2 µg/mL, Invitrogen) was used to detect bound biotinylated-Gal-8. Fluorescence was measured on a VICTOR^2^ plate reader (Perkin Elmer, Turku, Finland). Fifty microliters of each reagent diluted in 1% BSA-PBS was used per well. Each step was performed at 37 °C for 1 h and three washes with PBS-0.05% Tween were performed between the steps. All assays were carried out three times, each time in triplicates. Mouse laminin (10, 5, and 1 µg/mL), biotinylated-Gal-8, and 5% BSA were used as controls, and 100 mM lactose was added to biotinylated-Gal-8 for the inhibition studies.

### 2.7. SDS-PAGE and Gal-8 Blotting

SDS-PAGE was performed with 15 µL of each fraction (membrane preparation, wash, sucrose, lactose or pooled fraction U and B) was applied on NuPAGE gradient gel (Invitrogen, Grand Island, NY, USA) or Bio-Rad Mini-Protean TGX (4–20%). After electrophoresis, gels were stained with silver nitrate [27] or transfer blotted onto nitrocellulose (1 h 30 min at 30 mV for wet method or 7 min with I-Blot apparatus (Invitrogen)). Blots were probed with 1 µg/mL of biotinylated-Gal-8 followed by Strep-HRP (1:30,000, Vector Labs, Newark, CA, USA). All dilutions were made in TBST-BSA. Alternatively, Snap-ID (Millipore, Darmstadt, Germany) was used according to the manufacturer’s protocol. After three wash steps, membranes were developed as previously described with ECL. Negative control blotting was performed in parallel using only Strep-HRP.

### 2.8. Mass Spectrometry for Proteomics

Affinity chromatography was performed as indicated above, beginning the incubation with 8 mL of HL-60 membrane preparation with 3 mL of Gal-8-beads or Gal-8C-beads (10 mg/mL) in a 15 mL polypropylene tube under rotation for 1 h at 4 °C. The solution was poured onto a 10 mL glass wool column to trap the beads. Fractions were collected and electrophoresed as described above, and the Coomassie brilliant blue stainable bands of high molecular weight (75 kDa to 250 kDa) were excised. In-gel digestion with trypsin and liquid chromatography/tandem mass spectrometry (LC-MS/MS) sequencing were performed as described [28]. Peptide eluates were monitored in an MS survey scan followed by five data-dependent MS/MS scans on an LTQ-Orbitrap XL linear ion trap mass spectrometer (Thermo Fisher, San Jose, CA, USA). The Orbitrap was used to collect the survey MS scans (300–1600 *m*/*z*, 1,000,000 automatic gain control (AGC) target, 500 ms maximum ion time, resolution 60,000). The LTQ was used to acquire tandem MS/MS spectra (2 *m*/*z* isolation width, 35% collision energy, 5000 AGC target, 150 ms maximum ion time). Only ions with charge states 2+ or higher were selected for tandem MS. Dynamic exclusion was set to 30 s. The acquired MS/MS spectra were searched against a concatenated target-decoy human seq (release 54, 12 July 2012—34,421 target proteins) database of the National Center for Biotechnology Information using the SEQUEST Sorcerer algorithm (version 4.0.3, SAGE-N). Searching parameters included partially tryptic restriction, parent ion mass tolerance (±50 ppm), and dynamic modification of oxidized Met (+15.49 Da). The peptides were classified by charge state and tryptic state (fully and partial) and filtered dynamically by increasing XCorr and ΔCn values to reduce protein false discovery rate to less than 1%, according to the target-decoy strategy [29]. Peptide spectra and raw data have been uploaded on the PRIDE database (accession number PXD000664) [30]. Peptides and spectral counts as mass spectrometry parameters indicate the abundance of a particular glycoprotein. In our case, we were interested in glycoprotein candidates that may be involved in non-apoptotic PS exposure that seem to be localized to microdomains [6], therefore peptide abundances were not as relevant to our study.

### 2.9. Immunoblotting

Nitrocellulose membranes were blocked with TBS-5% milk and probed with mouse primary anti-CD45 (1:100, (clone 69 or clone HI30 biotinylated, BD Biosciences, Milpitas, CA, USA) or mouse primary anti-Basigin (1:1000, clone HIM6, Biolegend, San Diego, CA, USA) followed by HRP-conjugated goat anti-mouse secondary antibodies (1:5000, KPL, Sera Care, Milford, MA, USA) either overnight at 4 °C, 1 h at RT, or with Snap-ID (Millipore), with 3 washes with TBS-0.1% Tween between each incubation. Amount of antibody bound was revealed with ECL (SuperSignal West pico chemiluminescence, Thermo Fisher Scientific, Rockford, IL, USA) on an X-ray film (Genemate Blue autoradiography, Bioexpress, Kaysville, UT).

### 2.10. Cell Staining

HL-60 cells (100 µL of 10^6^/mL) in HANKS balanced media (HANKS) were incubated for 30 min on ice with 500 µL of anti-CD45-PerCy5.5 antibody (1 µg/mL, clone HI30, eBioscience, San Diego, CA, USA), washed with 3 mL of cold HANKS, centrifuged at 600× *g* for 7 min, resuspended in cold HANKS, and analyzed by flow cytometry (FACSCalibur, BD Biosciences, Milpitas, CA, USA) using CellQuest software v4.0.2 (BD Biosciences, Milpitas, CA, USA). Control staining was performed with 1 µg/mL of isotype control mouse IgG_1_K-PerCy5.5. HL-60 cells (100 µL of 10^6^/mL) in HANKS balanced media (HANKS), incubated 30 min on ice with 500 µL of mouse anti-human basigin-phycoerythrin antibody (1 µg/mL, clone 8D12, eBioscience, San Diego, CA, USA), following the same wash steps. Control staining was performed with 1 µg/mL of isotype control mouse IgG_1_K-phycoerythrin.

### 2.11. RT-PCR

Jurkat and HL-60 cells (10^6^ cells) were used for total RNA conversion using RNeasy mini kit (Qiagen, Germantown, MD, USA) following manufacturer’s instructions. PCR was carried out following Stanton and colleagues’ protocol [31] for CD45 isoforms (exon 2 and 7) and β-actin. Amplicons were electrophoresed 30 min on 1% agarose gel in Tris-Acetate Ethylene Diamine Tetraacetate (TAE) buffer with 1 μg/μL ethidium bromide and bands were visualized under ultraviolet light. Jurkat cells were used as a positive control, as they express different isoforms of CD45 [32].

### 2.12. Confocal Microscopy

HL-60 cells (500 µL of 10^6^ cells/mL) in HANKS were incubated 0.5 to 1 h on ice with 0.5 µg of anti-CD45-Alexa 488 (clone HI30, eBioscience or Biolegend), or with anti-basigin-Alexa 488 (clone HIM6, Biolegend), or with 0.5–0.25 µg Gal-8NM-biotin, or with Gal-8CM-biotin plus 1 µg Strep-Alexa 633 (Invitrogen). Controls were performed using fluorescently labeled mouse isotype control IgG_1_K-Alexa 488 (eBioscience) and with Strep-Alexa 633. After washing with 3 mL of cold HANKS and centrifugation at 600× *g* for 7 min, cells were resuspended in 100 µL cold HANKS and allowed to adhere on a cover-slip covered with poly-L-Lysine (BD Biosciences) for 30 min on ice. Cells were fixed with 1% paraformaldehyde in PBS overnight at 4 °C, washed three times in HANKS then mounted. Alternatively, cells were mounted in 1:1:0.01 (*v*/*v*/*v*) phosphate-buffered saline (PBS):glycerol:*p*-phenylenediamine, and visualized on a Zeiss LSM 510 Meta Confocal microscope (Carl Zeiss Microimaging, Thornwood, NY, USA) at the Epithelial Pathobiology Unit, Department of Pathology and Center for Neurodegenerative Disease at Emory University.

### 2.13. CD45 Inhibition

HL-60 cells (10^6^ cells/mL) were treated for 1 h 30 min with 1 µM of CD45 Phosphatase inhibitor N-(9,10-dioxo-9,10-dihydro-phenanthren-2-yl)-2,2-dimethyl-propionamide (CD45I, Calbiochem, San Diego, CA, USA) or vehicle (dimethyl sulfoxide, DMSO, Sigma, St. Louis, MO, USA), centrifuged at 600× *g* for 7 min, and re-suspended with Complete RPMI. Cells were incubated with Gal-8NM with or without 50 mM lactose or thiodigalactoside (TDG) for 4 h. Gal-8NM, R69H mutant of Gal-8, induced strong PS exposure on HL-60 cells [8]. PS exposure was analyzed with Annexin-V-Alexa 488 (Invitrogen) and propidium iodide by flow cytometry (BD FACSCalibur—CellQuest Pro v4.0.2, BD Biosciences) as previously described [8]. Statistical analysis was performed by Student’s *t* test using Microsoft Excel software v15. Prior to Western blot, HL-60 cells (10^6^ cells/mL) were pre-treated for 1 h with CD45I or DMSO, then with 5 µM Gal-8 with or without 100 mM lactose for 1 h. Cells were centrifuged at 600× *g* for 7 min, washed in cold PBS-100 mM lactose, lysed with 20 µL of Laemmli buffer [33], and then sonicated three times (3 s each). After 5 min centrifugation at 18,000× *g*, supernatants were boiled for 5 min, electrophoresed, and transblotted with I-Blot (Invitrogen), then probed overnight at 4 °C for ERK or phospho-ERK (p-ERK) with monoclonal rabbit antibodies (1:1000 in TBST-5% milk, clones 9101S and 9102, Cell Signaling, Danvers, MA, USA). After 3 washes in TBST and 1 h incubation at 37 °C with goat anti-rabbit-HRP (1:5000, KPL, Sera Care, Milford, MA, USA) in TBST-5% milk, bound antibodies were detected using ECL with Snap-ID as described previously. Total ERK was used as a loading control. Blots were reused after 5 min wash in the stripping buffer (65 mM Tris-HCl, pH 7.4, 2% SDS, and 100 mM BME), then incubated with anti-phospho-ERK antibody, then washed 5 min in TBS. After scanning the immunoblot films, Alpha Innotech software (AlphaView 3.0, San Leandro, CA, USA) was used to perform a relative quantification of p-ERK bands originating from Gal-8 treated samples, with and without lactose.

### 2.14. Inhibition of Glycosylation

HL-60 cells (10^6^ cells/mL) were treated with a potent inhibitor of mannosidase I, kifunensine (10 μg/mL, EMD Biosciences, Darmstadt, Germany) for 7 days, or with benzyl-αGalNAc, an O-glycosylation inhibitor, in DMSO (7.5 mM, EMD Biosciences) for 3 days. The cell media, containing freshly prepared inhibitors, was changed every day for benzyl-αGalNAc and every 1–2 days for kifunensine. Flow cytometry with lectin staining was used to confirm blockage of glycosylation. DMSO or water was added to the control flasks. Cells were spun at 600× *g* for 5 min, washed twice, then resuspended in HANKS-0.5% BSA (10^6^ cells in 500 μL). A volume of 100 μL of cell suspension was stained for 30 min with primary reactant (listed below) then with 1 μg of Strep-Alexa 488 or directly with mouse IgM anti-human CHO-131-FITC or mouse IgM isotype control-FITC (20 μL following protocols by Santa Cruz). Primary reactants were PHA-L-biotinylated (10 μg/mL, Vector Labs, Newark, CA, USA), ConA-biotinylated (1 μg/mL, Vector Labs, Newark, CA, USA), Gal-8NM-biotinylated (1 μg/mL), Gal-1-biotinylated (1 μg/mL), and Cholera toxin subunit B-biotinylated (10 μg/mL, Invitrogen). Cell suspensions were washed with 3 mL of HANKS-0.5% BSA, spun, and resuspended in 100 μL of HANKS-0.5% BSA between incubations. Staining was visualized by FACSCalibur (BD Sciences) and analyzed using CellQuest software v4.0.2 (BD Biosciences).

## 3. Results

### 3.1. Gal-8 Binds Specifically to a Subset of HL-60 Cell Surface Glycoproteins in a Carbohydrate-Dependent Fashion

**Figure 1:**
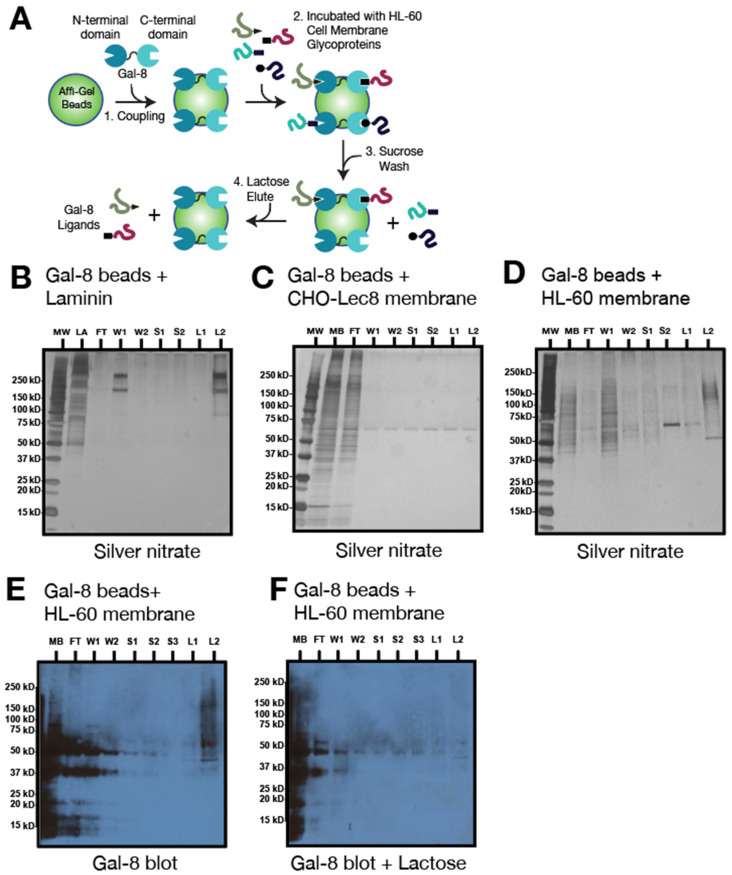
Validation of Gal-8 chromatography columns. (**A**) Schematic representation of Gal-8 chromatography with HL-60 cell membrane preparations and steps in the procedure. Gal-8 chromatography (1 mg Gal-8/mL beads) with (**B**), positive control: mouse laminin (LA); (**C**) negative control: CHO-Lec8 cell mutant membrane (250 μg/mL); (**D**) HL-60 cell membrane extract (250 μg/mL). (**B**–**D**) were visualized with silver nitrate staining. (**E**,**F**) Blotting of fractions from (**D**) with biotinylated Gal-8 (1 μg/mL) in absence (**E**) and presence (**F**) of lactose. Legend: MW, molecular weight markers; LA, laminin; MB, total membrane fraction; FT, flow through; W, wash; S, 100 mM sucrose wash; L, 100 mM lactose elution.

A variety of strategies have been employed in an effort to isolate the galectin receptors in various types of cells. Some studies have identified Gal-8 receptors on different cell types, such as on neutrophils (integrin alpha-M [34]) and HEK cells [35] (for a more extensive list, see [2]). In this study, we utilized lectin affinity chromatography on Gal-8 beads, followed by LC-MS/MS as a relatively straightforward approach that permitted the quantitative isolation of glycoproteins from HL-60 cells that are Gal-8 binding partners. To achieve this, we covalently coupled active Gal-8 to Affi-Gel-10 beads to create immobilized Gal-8 (Figure 1A). To ensure the binding and the specificity of Gal-8-Affi-Gel beads prior to the incubation with HL-60-derived material, we confirmed Gal-8 binding to control preparations, one positive control to which we expected Gal-8 to bind, and a negative control to which we expected no binding. For the positive control, we used mouse laminin, which is rich in polyLacNAc-containing glycans [36,37], and for the negative control, we used a membrane preparation of CHO-Lec8 cells, which lack galactose in N- and O-glycans due to a deficiency in a Golgi UDP-galactose transporter, and therefore lacks polyLacNAc epitopes [38]. As expected, laminin was detected in the lactose-eluted fractions, but not in those eluted by sucrose. In contrast, no protein material could be eluted with lactose when the CHO-Lec8 cell membrane preparations were chromatographed on Gal-8 beads (Figure 1B,C). These data confirm that the Gal-8-Affi-Gel was active and capable of binding glycoproteins Gal-8 ligands. For examining Gal-8 receptors in HL-60 cells, we first used a membrane preparation of these cells and stained the eluted proteins in all fractions by silver nitrate (Figure 1D). Membrane glycoproteins from HL-60 cell specifically eluted by lactose (lane L2) ranged from ~50 to ~250 kDa. A similar profile was detected for the lactose-eluted fractions (L2) after blotting with biotinylated Gal-8 (lane L2, Figure 1E). This binding was abolished by incubation of biotinylated Gal-8 with lactose, an inhibitor of Gal-8 binding (lane L2, Figure 1F).

**Figure 2:**
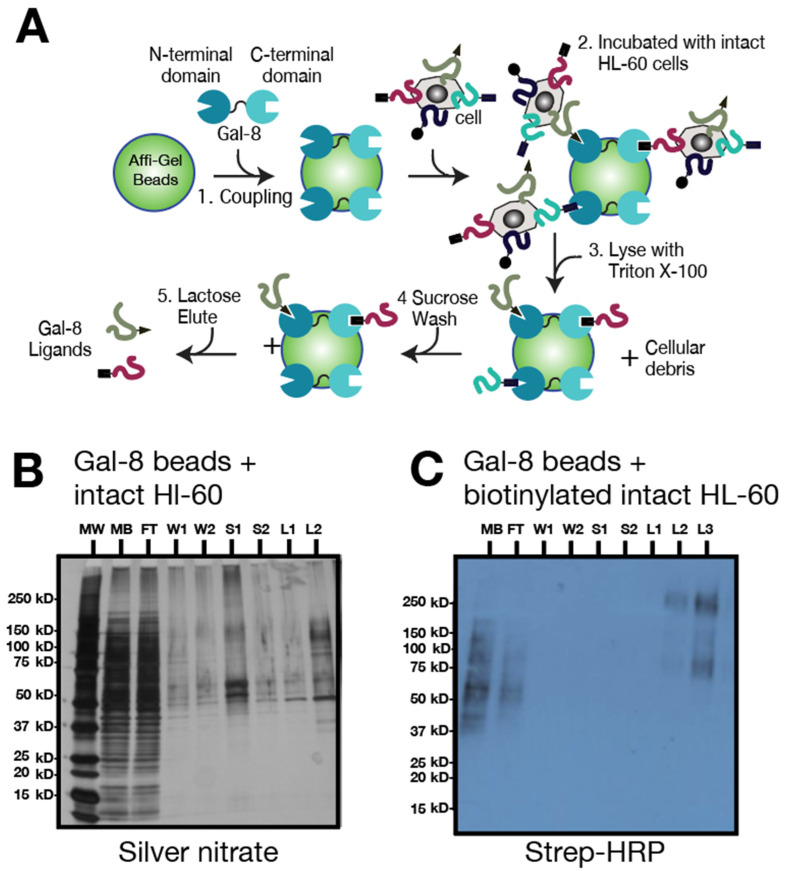
Gal-8 chromatography with intact HL-60 cells. (**A**) Schematic representation of Gal-8 chromatography steps with intact HL-60 cells. (**B**) Gal-8 chromatography (1 mg Gal-8/mL beads) of intact, cell surface biotinylated HL-60 cells. Samples were visualized with silver nitrate staining. (**C**) Strep-HRP blot (1 μg/mL) of fractions from B with intact biotinylated HL-60 cells. Legend: MW, molecular weight markers; MB, total membrane fraction; FT, flow through; W, wash; S, 100 mM sucrose wash; L, 100 mM lactose elution.

As a second approach, and to confirm the presence of Gal-8 binding partners among the HL-60 cell surface glycoproteins, we incubated immobilized Gal-8 with either intact HL-60 cells or surface-biotinylated HL-60 cells. HL-60 cells bound to the Gal-8 beads were lysed with TX and the biotinylated glycoproteins were washed with sucrose (fractions S1 and S2), eluted with lactose (in three fractions, L1–L3), and analyzed by Strep-HRP assay (Figure 2A). Bound glycoproteins detected by Strep-HRP showed a similar profile (ranging from ~75 kDa to ~250 kDa) to that seen with HL-60 cells upon chromatography that were stained by silver nitrate, indicating that such glycoproteins are located at the cell surface (lane L2, Figure 2B and lane L3, Figure 2C).

**Figure 3:**
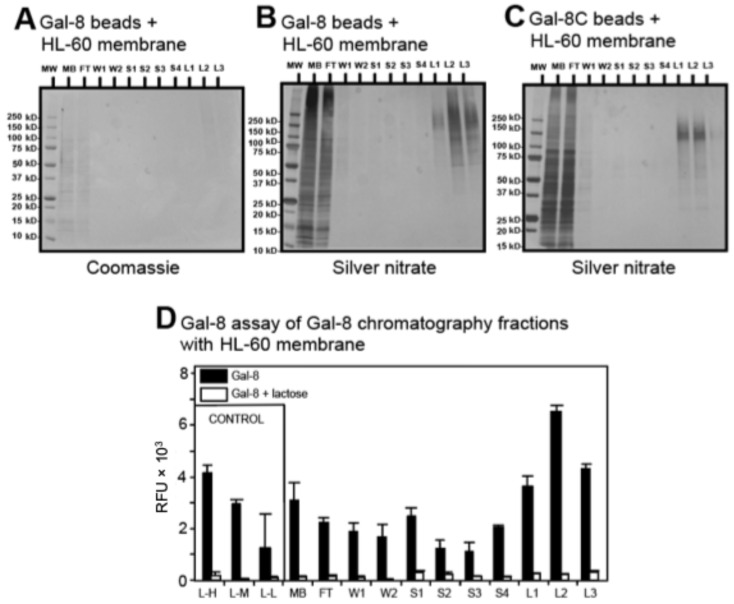
Gal-8 binds to specific sets of glycoproteins. (**A**,**B**) High-density (10 mg Gal-8/mL beads) full-length Gal-8 chromatography with 8 mL of HL-60 cell membrane extract (250 μg/mL) visualized by Coomassie brilliant blue staining and silver nitrate, respectively; (**C**) high-density (10 mg Gal-8C/mL beads) Gal-8C domain chromatography with 8 mL of HL-60 cell membrane extract (250 μg/mL) visualized by silver nitrate; (**D**) Gal-8 binding to protein fractions eluted from Gal-8 chromatography fractions with HL-60 cell membrane preparation. Results represent mean of 2 independent experiments performed in triplicate. Error bars represent means ± 1 standard deviation (SD). Legend: MW, molecular weight markers; MB, total membrane fraction; FT, flow through; W, wash; S, 100 mM sucrose wash; L, 100 mM lactose elution; L-H, laminin at high amount (10 μg); L-M, laminin at medium amount (5 μg); L-L, laminin at low amount (1 μg).

To be able to detect and identify the glycoproteins that are binding partners of immobilized Gal-8, we combined the affinity chromatography with Gal-8 beads and LC-MS/MS. In brief, we scaled up the amounts of HL-60 cell membrane preparations and the amount of immobilized Gal-8 on Affi-Gel. With this method we could obtain Coomassie brilliant blue-detectable bands after SDS-PAGE of lactose-eluted fractions, which we subjected to tryptic digest followed by LC-MS/MS (Figure 3A). As it can be difficult to stain heavily glycosylated proteins by Coomassie blue because of glycan moiety interference [39], we used in parallel silver nitrate to confirm Coomassie staining (Figure 3B). Because the C-terminal domain of Gal-8 was shown to have signaling activity in HL-60 cells [8], we compared the binding of HL-60 cell glycoproteins to the recombinant Gal-8C domain (Gal-8C-Affi-Gel) (Figure 3C) to that of full-length immobilized Gal-8. We excised the high molecular weight (~75 kDa-250 kDa) Coomassie blue-stained bands from the lanes originating from the lactose-eluted fractions L1–L3 (Figure 3A). We did the same for Gal-8-Affi-Gel and Gal-8C-Affi-Gel chromatography and all of the protein samples were subjected to identification by LC-MS/MS. 

To assay the efficiency of isolation in our chromatographic approach, we developed an ELISA-type assay for semi-quantitative detection of proteins bound to immobilized Gal-8 employing biotinylated Gal-8 followed by staining with Strep-Alexa 488 (Figure 3D). The results are expressed as relative fluorescence units (RFU) according to the chromatography fractions. Maximal fluorescence was detected for the lactose-eluted fractions with more than 3 × 10^3^ RFU, close to positive control fluorescence levels for immobilized mouse laminin (10 µg of mouse laminin, 4 × 10^3^ RFU), and at nearly twice that of the negative control (sucrose eluted fraction number 1.2 × 10^3^ RFU). The binding of Gal-8-biotin was inhibited incubation with 100 mM lactose (white bars, Figure 3D), demonstrating that binding required carbohydrate recognition. These results indicate that most of the Gal-8 chromatography with binding glycoproteins in HL-60 cell extracts identified by affinity chromatography, as assessed by this ELISA-type assay, are found in the lactose-eluted material.

### 3.2. Cell Surface Glycoproteins Captured on Immobilized Gal-8C Are a Subset of the Total Glycoproteins Captured by Immobilized Gal-8

Table 1 lists the top twenty proteins bound by immobilized Gal-8 and eluted with lactose, as identified by LC-MS/MS. Appendix A (Supplemental Table S1) contains a full list of all 146 proteins identified by LC-MS/MS. Importantly, only 13 of the 20 proteins overlapped between immobilized Gal-8 and Gal-8C. Thus, HL-60 cell surface glycoproteins captured on the immobilized Gal-8C are a subset of those that bound to the immobilized full-length Gal-8. As expected, the glycoproteins eluted from the immobilized Gal-8 and Gal-8C and identified by LC-MS/MS, and the glycoproteins detected by silver nitrate staining of electrophoretically-resolved protein bands had comparable molecular weights (~75–250 kDa) (Figure 3A–C). Eighteen of these detected glycoproteins are cell surface glycoproteins that can be divided into three groups: (group 1) enzymes; cytochrome B-245, membrane metalloproteases (CD13, leucyl-cystinidyl aminopeptidase, carboxypeptidase D), phosphatases (CD45 isoform A and C); (group 2) transporters (CD98, ATP-binding cassette 1 and 4, transferrin receptor, ATPase Na^+^/K^+^, Na^+^/HCO_3_^−^ cotransporter, neutral amino acid transporter); and (group 3) potential signaling proteins (CD147, CD43, and the integrins CD49d, CD51 and CD29). While CD45RC was identified in fractions eluted from immobilized Gal-8 and Gal-8C, CD45RA was only found in the full-length Gal-8, which may indicate that it bound to the N-terminal domain of Gal-8 possible binding to Gal-8 N-terminal. CD13 was among the most abundant proteins identified by mass spectrometry on affinity purified materials using both full-length Gal-8 and C domain (Table 1). The remaining two proteins are found in endosomes/cytoplasmic granules (hornerin and ATPase/H^+^). The presence of hornerin may be due to contamination during membrane preparation, as it is typically found in epidermal cells [40]. However, it has been shown to be expressed at the cell surface of other cells such as endothelial [41,42] and extracellular matrix of breast cancer cells [43]. As HL-60 are a myelocytic cancerous cell line, they might also be present at the cell surface in these cells. ATPase, typically found in the lysosome, can be found in plasma membrane of cancerous cells [44,45].

### 3.3. CD45 Is Involved in Non-Apoptotic PS Exposure Induced by Gal-8

**Figure 4:**
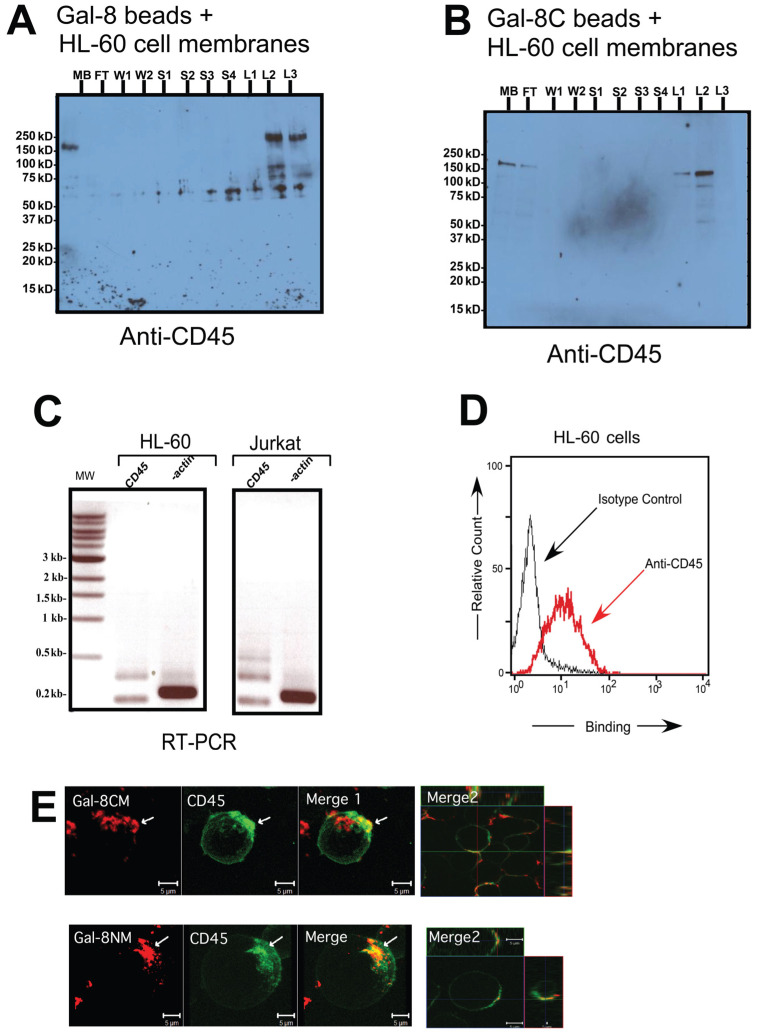
CD45 was detected among the Gal-8 binding proteins on HL-60 cells. (**A**) Anti-CD45 blot of full-length Gal-8 chromatography with HL-60 cell membranes; (**B**) anti-CD45 blot of Gal-8C chromatography with HL-60 cell membranes; (**C**) Reverse Transcriptase-PCR of CD45 isoforms and beta-actin with HL-60 and Jurkat cells. Specific amplicons for CD45 (about 200, 350, and 500 bp) are visible; (**D**) HL-60 cell staining with anti-CD45-Alexa 488 or isotype control measured by flow cytometry; (**E**) confocal images of HL-60 cells treated at 4 °C with biotinylated Gal-8CM (**top row**) or Gal-8NM (**bottom row**) with Strep-Alexa 633 (red) and anti-CD45-Alexa 488 (green); Merge 1: whole cell reconstruction, Merge 2: detailed colocalization (yellow), scale bar = 5 μm. White arrows indicate colocalization in punctate membrane microdomains. Legend: MB, total membrane fraction; FT, flow through; W, wash; S, 100 mM sucrose wash; L, 100 mM lactose elution; MW, molecular weight.

The presence of CD45 in the cell membrane preparation of HL-60 cells in the lactose eluates from the immobilized Gal-8 (L2, L3) and Gal-8C (L1, L2) was confirmed by immunoblot using anti-CD45 antibody (Figure 4A, lane L2–L3 and Figure 4B, lane L1–L2). RT-PCR experiments showed that two isoforms of CD45 transcripts were expressed in HL-60 cells (middle lane in Figure 4C). Flow cytometry experiments confirm the presence of CD45 on the cell surface of HL-60 cells (Figure 4D). These data are in accordance with previous data on DMSO-treated HL-60 cells [46] and myelocytic cells [47] showing elevation of CD45 in differentiated cells. Confocal microscopy was employed to show the distribution of CD45 on the cell surface, in which we observed that CD45 appeared to be distributed all around, but more concentrated in a pole where it co-localized with Gal-8 on the cell surface (Figure 4E).

**Figure 5:**
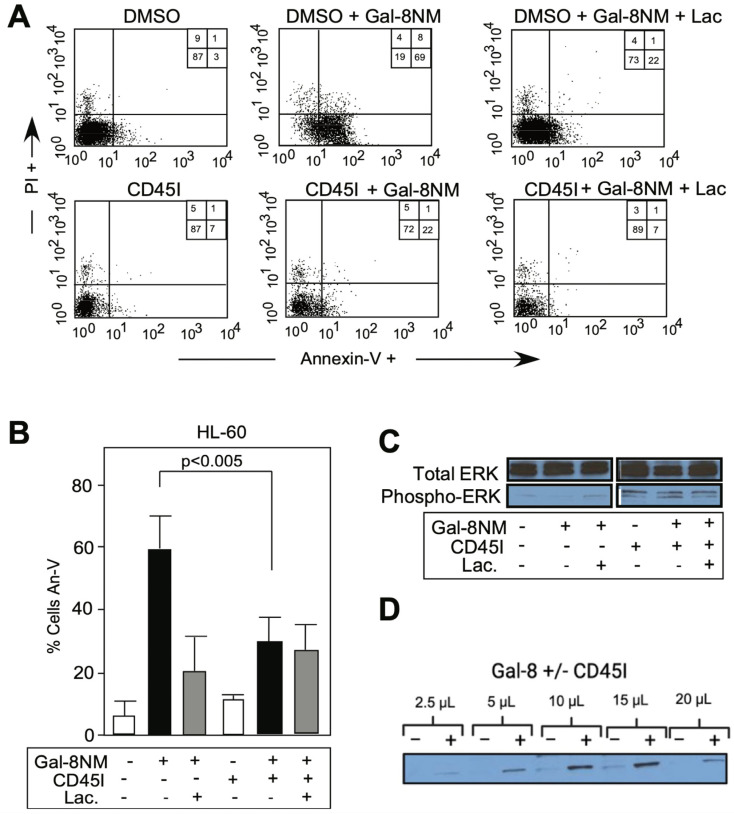
Gal-8-induced phosphatidylserine (PS) exposure is blocked by inhibition of CD45 phosphatase activity. (**A**) Flow cytometry dot plot of HL-60 cells treated with 0.6 μM Gal-8NM final with or without 100 mM lactose (Lac) in presence of vehicle (DMSO) or CD45 inhibitor (CD45I, 1 μM); Cells were stained with Annexin-V (An-V) and propidium iodide (PI) to measure PS exposure (Annexin-V positive/PI negative); (**B**) PS exposure quantification of three independent experiments in duplicate of panel (A), *p* < 0.005 between DMSO-treated and CD45I-treated samples. Error bars represent means ± 1 SD of three independent experiments in duplicate; (**C**) Western blot detection of phospho-ERK and total ERK after 1 h incubation of HL-60 cells with Gal-8NM with or without lactose, and with DMSO or CD45 inhibitor (CD45I); (**D**) Western blot detection of p-ERK after 1 h incubation with Gal-8 with (+) or without (−) CD45I with increased loading of HL-60 cell lysate (2.5 to 20 μL).

To test whether CD45 plays a role in Gal-8-induced PS exposure, we challenged cells with a specific inhibitor of CD45 phosphatase activity, CD45I. Figure 5A depicts the flow cytometry assay of HL-60 cells after annexin V staining. We observed that the majority of HL-60 cells failed to expose PS in the presence of CD45I when stimulated with Gal-8NM for 4 h (CD45I+Gal-8NM in Figure 5A) in comparison to the cells stimulated by Gal-8NM without CD45I (DMSO+Gal-8NM). Gal-8NM, an R69H mutant of Gal-8, is known as a strong inducer of PS exposure on HL-60 cells [5]. There was a statistically significant difference between control cells (DMSO-treated) and CD45I-treated cells, when both cell groups were co-incubated with Gal-8NM (*p* < 0.05, Figure 5B). The number of HL-60 cells that exhibited a non-apoptotic PS exposure after being subjected to CD45I (CD45I in Figure 5A) was significantly smaller when compared to the control cells subjected to DMSO (DMSO in Figure 5A). The specificity of the CD45 inhibition of PS exposure induced by Gal-8 binding to its glycoprotein partners was tested by the effects of lactose. The inhibition could be reversed by lactose, due to Gal-8NM binding to glycan determinants on CD45. It is known that in other types of cells, Gal-8 ligation to cell surface glycoproteins can trigger the ERK signaling pathway, after the phosphorylation of ERK into p-ERK by the action of specific kinases [48]. Hence, we hypothesized that the level of p-ERK will mirror the extent of Gal-8 ligation of cell surface glycoproteins, including CD45, leading to PS exposure. As shown by Western blot, Gal-8-induced PS exposure was inhibited by CD45I. This inhibition appears to increase p-ERK levels (Figure 5C,D). Hence, Gal-8-induced PS exposure may involve a pathway that involved ERK signaling, either directly or indirectly.

p-ERK blotting indicated that ERK might act as a direct substrate of CD45 after activation, as more p-ERK was observed after CD45 inhibition. Our findings are concordant with those of others [49] who demonstrated that CD45 was able to dephosphorylate p-ERK, thus acting as a player in the ERK signaling pathway. We have shown herein that Gal-8 inhibited ERK pathways on HL-60 cells, contrary to Gal-8 effect on Jurkat cells [50] or platelets [51], but similarly to the effect of Gal-3 on retinal pigment epithelial cells [52].

**Figure 6:**
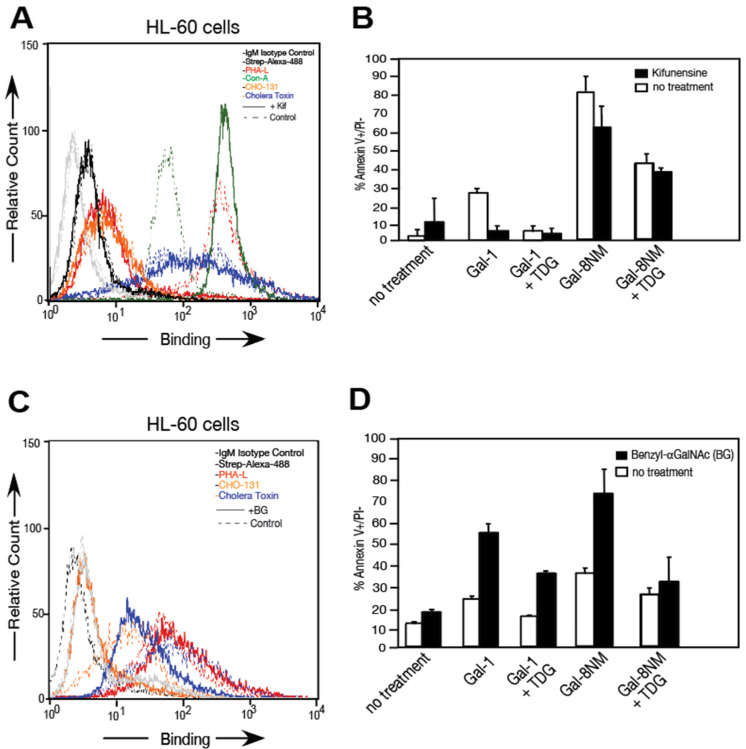
Gal-8NM signaling is not inhibited by kifunensine (Kif) nor benzyl-αGalNAc (BG). (**A**,**C**) HL-60 cell staining with PHA-L (red), ConA (green), CHO-131-Alexa 488 (orange), Cholera toxin subunit B (blue), isotype control (grey), and Strep-Alexa 488 (black) measured by flow cytometry in presence or absence of inhibitors A: Kif or C: BG (solid lines) or absence (dotted-lines). (**B**,**D**) PS exposure quantification of three independent experiments in duplicate with Gal-8NM and Gal-1 in the presence of inhibitors (**B**): Kif or (**D**): BG (black) or absence (white). Error bars = means ± 1 SD.

As CD45 bears N- and O-glycans, we used reagents to further characterize the importance of the interactions between these glycan-binding proteins and the types of glycans. We utilized an inhibitor of N-glycosylation, kifunensine [53] and an inhibitor of O-glycosylation, benzyl-αGalNAc [54]. Our group previously characterized a monoclonal antibody, CHO-131, with specificity for core 2 O-glycans [55], and we used the widely known cholera toxin which binds to gangliosides [56]. Plant lectins were also used, including concanavalin A (ConA), which preferentially binds high mannose and hybrid N-glycans and some biantennary N-glycans [57], and phytohemagglutinin-A (PHA-L), which binds tri- and tetra-antennary N-glycans [58]. In kifunensine-treated samples as well as control samples, the binding of CHO-131 antibody and cholera toxin were not affected by kifunensine. ConA binding was stronger for the kifunensine treated samples whereas binding PHA-L was almost abolished (Figure 6A). In the presence of kifunensine, Gal-1 treatment caused a significant decrease in PS exposure contrary to Gal-8NM-treated samples when compared to the controls (Figure 6B). In benzyl-αGalNAc-treated samples as well as control samples, PHA-L binding did not change; a slight decrease of cholera toxin binding was observed and CHO-131 binding was almost abolished (Figure 6C). In the benzyl-αGalNAc-treated samples, Gal-1 and Gal-8NM caused a substantial increase in PS exposure, which was carbohydrate-dependent and partly blocked by including the hapten inhibitor TDG (Figure 6D).

### 3.4. Gal-8 Binding to Basigin (CD147) Depends on the polyLacNAc Moieties on N-Glycans of CD147

**Figure 7:**
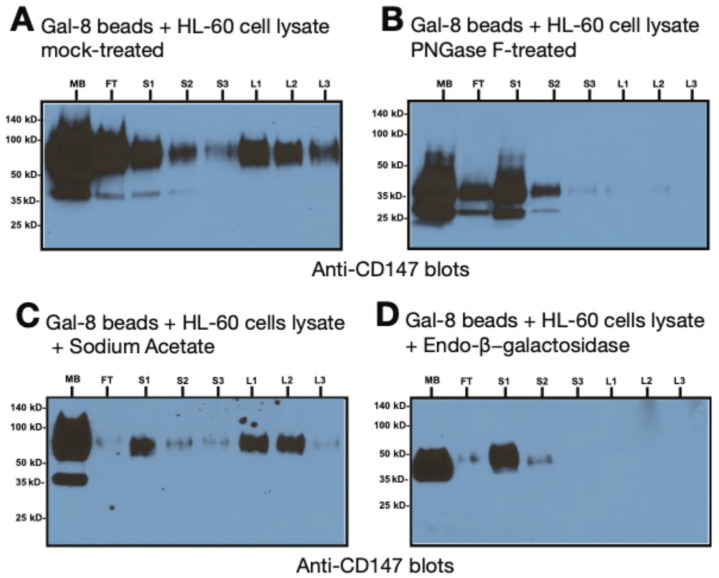
Gal-8 binds to basigin through polyLacNAc present on N-glycans. (**A**) Gal-8 affinity chromatography of HL-60 cell membrane preparations. Blots were stained with anti-basigin (CD147) antibody, followed by HRP-conjugated goat anti-mouse, followed by ECL. (**B**–**D**) Anti-basigin blot of full-length Gal-8 chromatography with HL-60 cell membranes; washes were performed with 3 mL of 100 mM sucrose (S) and elution with 3 mL of 100 mM lactose in PBS-TX; (**A**) mock-treated with buffers alone or (**B**), PNGase F-treated; (**C**) mock-treated with sodium acetate or (**D**), treated with endo-β-galactosidase (*E. freundii*). Legend: MB, total membrane fraction; FT, flow through; S, 100 mM sucrose wash; L, 100 mM lactose elution.

Basigin was detected by immunoblotting with mouse anti-basigin antibodies on the lactose eluted fractions L1 to L3 from the mock-treated lysate with molecular weights ranging from 50 kDa to 100 kDa (lane L1, L2, L3, Figure 7A). PNGase F treatment of HL-60 cell preparation to broadly remove most N-linked glycans from glycoproteins [59] did not produce any lactose-eluted material (lane L1, L2 and L3, Figure 7B). In this PNGase F treated HL-60 cells lysate, a size reduction can be noticed, with basigin now ranging from 50 kDa to 25 kDa (lane MB, Figure 7B). This result suggested that HL-60 basigin is N-glycosylated and the N-glycans are essential for Gal-8 binding. Basigin is found in the lactose-eluted fractions L1 and L2 from the mock-treated sample with sodium acetate (lane L1, L2, Figure 7C) but is absent on the lactose-eluted fractions from the endo-β-galactosidase treated material (lane L1, L2 and L3, Figure 7D), which hydrolyzes internal β1-4-galactose linkages within a variety of structures. The size of the high molecular glycoform of basigin shifted from 100 to 50/35 kDa on this endo-β-galactosidase treated starting material (lane MB, Figure 7D). Thus, basigin appears to express polyLacNAc glycan epitopes that are essential for Gal-8 binding. In total, these results suggest that Gal-8 binding to HL-60 basigin is strongly dependent on polyLacNAc glycans specifically found on the N-glycans.

### 3.5. Gal-8 Binds to Basigin (CD147) on Both Domains and Colocalized on HL-60 Cells

**Figure 8:**
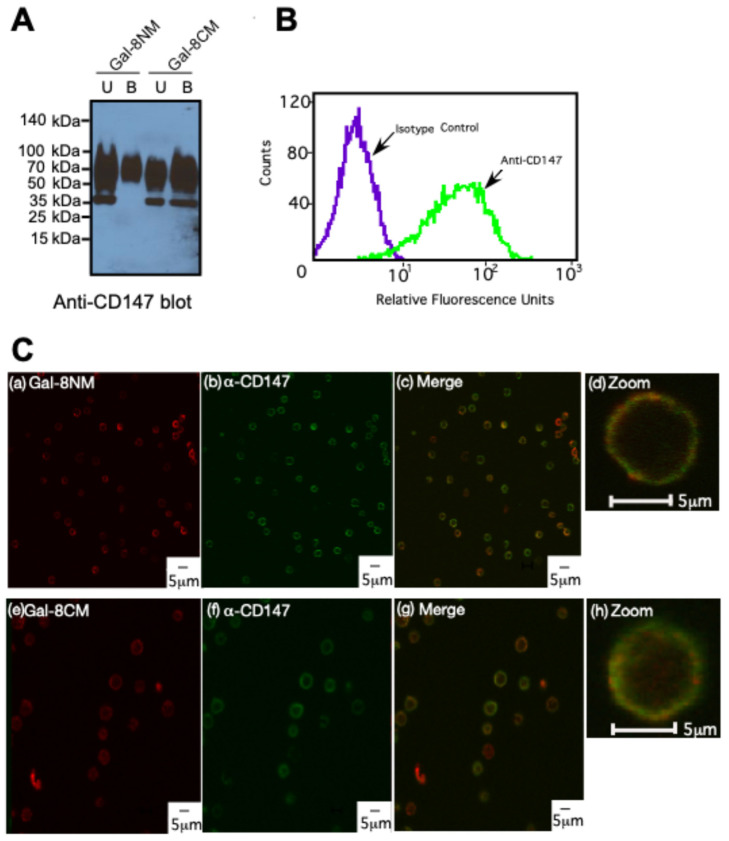
Both N- and C-terminal domains of Gal-8 bind to basigin and colocalize with basigin on HL-60 cells. (**A**) Anti-basigin (CD147) blot of Gal-8NM and Gal-8CM chromatography with HL-60 cell membranes (U: Unbound, B: Bound); (**B**) HL-60 cell staining with anti-basigin-Alexa 488 or isotype control measured by flow cytometry; (**C**) confocal images of HL-60 cells treated at 4 °C with (a) biotinylated Gal-8NM or (e) Gal-8CM with Strep-Alexa 633 (red) and (b,f) anti-basigin-Alexa 488 (green); Merged images Gal-8NM/anti-basigin and Gal-8CM/anti-basigin are shown (c,g), and zoomed images on two individual cells from (c,g) are shown (d,h), respectively. Scale bar represents 5 μm.

To address the question of which domain of Gal-8 is responsible for the interaction with basigin, we separately coupled the Gal-8 N- and C-domain mutants on Affi-Gel-10 columns, then pooled flow through and sucrose washed fractions (U, unbound) and lactose eluted fractions (B, bound) from HL-60 cell lysate. Basigin was detected in bound fractions for both domains (Figure 8A). The only difference was that the C-domain mutant (i.e., the Gal-8 N-domain) binds a lower (~35 kDa) molecular weight isoform of basigin, which is likely the high mannose-type N-glycan version of basigin [60]. Basigin is expressed at the cell surface of HL-60 cells (Figure 8B), as seen in flow cytometry, and colocalized with binding of both domains of Gal-8 (Figure 8C), as observed by confocal fluorescence microscopy. No binding was observed in our controls with Strep-Alexa688 and mouse isotype control IgG-Alexa 488. Therefore, both CRDs of Gal-8 appear to bind basigin from HL-60 cells.

## 4. Discussion

In this study, using a systematic biochemical and proteomic approach, we identified CD45 as a functional ligand for Gal-8-induced *preaparesis*, which is the induction of PS exposure independently of apoptosis, in leukocytes [8,10,11]. We first identified ligands by an affinity chromatography approach followed by proteomics, then confirmed the presence of CD45 as a ligand for Gal-8 by flow cytometry and confocal microscopy, and blocked its activity to assess its role in Gal-8-induced PS exposure. Our findings are consistent with those obtained through glycoproteomics approaches used by Mitsui et al. [61] who employed a polyLacNAc binding lectin *Datura stramonium* agglutinin (DSA) on immune cancerous cell lines such as Jurkat and U937. They identified CD45, as well as CD147 and CD98 [61]. Togayachi et al. [62] used hydrophilic interaction chromatography (HILIC) to isolate polyLacNAc containing glycoproteins on HL-60 cells (see Appendix A, Supplemental Table S1).

As noted in Table 1, CD13 was among the most abundant proteins identified by MS using both full-length Gal-8 and C domain. CD13 acts as a receptor for coronavirus, and was shown to be involved in adhesion and apoptosis [63]. Whereas it contains putative O- and N-glycosylation sites [64], there are few studies on its glycosylation, and no antibodies that would block glycan attachment are available. This fact led us to explore whether inhibition of the metalloprotease activity of CD13 by bestatin would block Gal-8-induced PS exposure and *preaparesis*. We observed that blocking CD13 activity with bestatin had no effect on *preaparesis* in HL-60 cells and we did not pursue that further.

We then focused on CD45, which was bound to both Gal-8 and the Gal-8C domain. There were several reasons for pursuing studies on CD45. CD45 from leukocytes is known to carry N- and O-glycans with the former possessing polyLacNAc motifs [65]. We have previously reported that Gal-8 binds well to polyLacNAc-containing glycans [8]. Moreover, other members of the galectin family, such as galectin-1, can bind to CD45 [66]. Furthermore, CD45 has different isoforms depending on the presence of short extra-peptides which possess different amounts of O-glycans [47]. These glycoforms are known as CD45R0, RA, RB, RC, and RABC, and are generated due to splicing. The molecular weights of CD45RO, RB, RA, RBC, and RABC are 180, 190, 210, and 230, respectively. Each N-glycan is complex type. RO has core 2 O-glycans; RB, RA, and RBC have sialylated core 1 O-glycans. All N-glycan sites are occupied in all isoforms; some O-glycan sites may overlap between isoforms, but the total number of O-glycans should be higher for larger isoforms (i.e., RO should have the least number of O-glycans, while RABC should have the most O-glycans). Different isoforms are expressed on different type of leukocytes and stages of maturation, and may control leukocyte activation [47].

We observed Gal-8 domain specificity for binding of CD45 based on our proteomics data. CD45RC, but not CD45RA, was found to bind to immobilized Gal-8C, which is the signaling domain for *preaparesis* (Table 1). CD45 was characterized as a negative regulator of PS exposure on B cells and thymocytes [24,67] and is involved in neutrophil regulation/activation [68]. CD45 phosphatase activity can be blocked by a specific inhibitor [50,69,70]. For all of these reasons we chose to focus on CD45 as a possible candidate involved in Gal-8-induced PS exposure in HL-60 cells. Similar CD45 distribution has been observed at the cell surface of leukocytes [71,72]. CD45 and Gal-8 colocalized on a polarized region of the cell surface where CD45 is enriched at the leading edge (Figure 4E). This could reflect the presence of specific glycans on CD45 at this pole in comparison to the rest of the cell. In contrast to the methods of Cattaneao et al. [73], we performed our confocal microscopy at 4 °C where lateral diffusion of membrane proteins are prevented. Therefore, galectin receptors seemed to be already present at punctate domain of the cell. It has been reported that during neutrophil activation, fusion of granules carrying CD45 with the cell membrane occurs [74], which further emphasizes the potential importance of CD45 during neutrophil activation. Our data suggest the potential importance of CD45 in Gal-8-induced PS exposure, as PS exposure was reduced through direct inhibition of CD45 phosphatase activity (Figure 5).

CD45 was also detected on activated human neutrophils and bound to the Gal-8C domain. In our experiments, we used Gal-8NM, a Gal-8 isoform carrying an R69H mutant in the N-terminal domain, which induced strong PS exposure on HL-60 cells [8]. Whereas Gal-1-induced PS exposure was decreased when high mannose, hybrid, and complex-type N-glycans were reduced by kifunensine treatment, Gal-8NM still signaled PS exposure (Figure 6A,B). The inhibition of O-glycan synthesis in HL-60 cells by benzyl-αGalNAc caused an increase in PS exposure when cells were treated with Gal-1 or Gal-8NM (Figure 6C,D). It is possible that both N- and O-glycans are required for Gal-8NM signaling. We used both inhibitors, kifunensine and benzyl-αGalNAc, in the same sample, but inhibition of O-glycans was not complete, and therefore we could not make a definitive conclusion in that direction. As a control, we also inhibited glycolipid synthesis in HL-60 cells with different concentrations (1 to 10 μM) of 1-phenyl-2-decanoylamino-3-morpholino-1-propanol, but our trials were unsuccessful because of cell toxicity of the drug at high concentrations and weak inhibition observed at lower concentrations. In light of our finding from inhibition of CD45 phosphatase activity and the inhibition of N- and O-glycan synthesis, we conclude that CD45 plays an important role in Gal-8-induced non-apoptotic PS exposure. It may be that other ligands identified by our proteomics approach in discovering Gal-8 binding partners are involved in the process leading to PS exposure. For instance, recent data suggested that cross-linking antibodies to CD13 on another leukocytic cell line (U937) resulted in pro-apoptotic signaling including PS exposure [63]. It might be that protein complexes are involved in the signaling leading to PS exposure (galectin/glycoprotein/protein interactions) that cannot be fully resolved only by proteomic approach or inhibition assays. This is the case for the new Gal-8 ligands that we identified for the first time, which included CD98 and CD147, cell surface glycoproteins that can form complexes with integrins [75,76,77].

A key novel discovery in our work was the finding that Gal-8 interacts with basigin/CD147 in a carbohydrate-dependent manner (Figure 7A–D). The amino acid sequence of CD147 predicts a protein of only 29 kDa, with three consensus N-glycosylation sites. Assuming these glycans are large in size, bearing multiple polyLacNAc chains, their total molecular mass could increase the molecular weight of CD147 to the observed range of 35 kDa to 64 kDa [78]. Consistent with this possibility, previous studies identified two main glycoforms of basigin: highly glycosylated (64 kDa) and sparsely glycosylated (35 kDa) [60,79]. In the present study we revealed that Gal-8 binding to the highly glycosylated form of CD147 in a carbohydrate-dependent manner; the latter could be eluted by lactose. Enzymes that strip away N-glycans (PNGase F) or enzymes that cleave within N-glycans (endo-β-galactosidase) interfered with the Gal-8 binding to basigin (Figure 7). Considering that endo-β-galactosidase, which specifically cleaves polyLacNAc chains [80], significantly reduced the high molecular weight glycoform of basigin to ~50 kDa, assuming a lactosaminyl unit is ~374 Da, it may be speculated that the high molecular weight glycoform of basigin may possess many dozens of repeated lactosaminyl units on its N-glycans. This is consistent with other reports on the presence of long chain polyLacNAc repeats on N-glycans attached to cell surface glycoproteins [81,82,83]. We also previously reported on long chain polyLacNAc in O-glycans of glycoproteins in HL-60 cells [84].

CD147 has many aliases—basigin (basic immunoglobulin superfamily) [85], leukocyte activation antigen M6 [78], OK blood group [86], or EMMPRIN (extracellular matrix metalloprotease inducer) [87]. CD147 also has many binding partners—Gal-3 [25], caveolin-1 [60], cyclophilin [88,89], α_3_β_1_ integrin [90], monocarboxylate transporter CD98 [75,76], E-selectin [79], and XK-related protein 8 (Xkr8) [91,92,93]. Interestingly, Gal-8 is also known to bind several integrins, including α_3_β_1_, which is important in cell adhesion [94,95]. Xkr8a is a plasma membrane phospholipid scramblase, whose role is facilitating phospholipid movement across two plasma membrane leaflets and which is highly expressed in neutrophils and HL-60 cells. It should be emphasized that Xkr8a is important in PS exposure in apoptosis [92,96,97]. We speculate that Gal-8, via its interaction with basigin, directly activates Xkr8 through basigin interactions.

## 5. Conclusions

**Figure 9:**
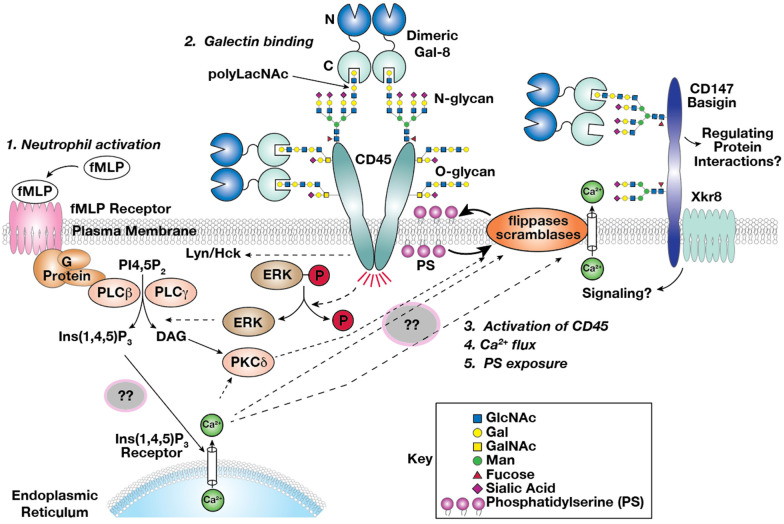
Working model of Gal-8-induced PS exposure on neutrophils. (1) Neutrophils can be activated after formyl-Methionyl-Leucyl-Phenylalanine (fMLP) receptor stimulation by degraded bacterial or mitochondrial proteins from dying surrounding cells; (2) Dimeric Gal-8 expressed by endothelial cells (ECs) or smooth muscle cells (SMCs) binds to polyLacNAc containing glycoproteins, particularly CD45, (3) which is upregulated during neutrophil activation [47]. CD45 signals through its phosphatase activity, dephosphorylating directly or indirectly phospho-proteins such as p-ERK, (4) leading to an increase of cytoplasmic calcium ions (Ca^2+^). This cation increase may be a synergistic result with fMLP receptor signaling through G-Proteins, which also conduct Protein kinase delta activation (PKCδ). PKCδ and Ca^2+^ will then (5) activate scramblase, which results in PS exposure [13,97]. Synergistically, dimeric Gal-8 could also bind to a CD147-Xkr8 complex, and possibly directly activate scramblase activity by Xkr8 [91]. ?? represents an unknown component of the process.

Based on finding presented herein and in our earlier studies and previous inhibition studies with Gal-1 and neutrophils [98], we put forward a working model of Gal-8 interactions with CD45 and basigin (Figure 9). (**1**) After the activation of neutrophils, (**2**) dimeric Gal-8 binds polyLacNAc chains on N-glycans and O-glycans belonging to CD45, thus activating CD45, (**3**) which triggers a signaling cascade involving direct or indirect dephosphorylation of ERK and other kinases. It is known that Gal-8 ligation to respective receptors can trigger many signaling pathways including the ERK pathway [48]. This dephosphorylation cascade may (**4**) respond to an increase in the flux of calcium ions, thus (**5**) affecting PS exposure enabled via scramblase. Gal-8 could bind to CD147 on activated neutrophils, thus increasing the activity of the phospholipid scramblase xkr8 [92,97], which leads directly to the PS exposure. It is well known that PS exposure is an ‘eat me’ signal for activated neutrophils, causing them to be recognized by phagocytotic cells and taken up by macrophages and other cells, which can lead to the resolution of inflammation at sites of infections [10,13]. Our working model suggests additional studies to further explore the interactions of Gal-8 with membrane glycoproteins and the complex signaling pathways induced.

## Figures and Tables

**Table 1:** List of HL-60 cell surface glycoproteins specifically eluted from the immobilized Gal-8 or Gal-8C as identified by LC-MS/MS. Identified proteins using both the full-length and the C domain of Gal-8 are in bold, and proteins found only with full-length Gal-8 are in italics. Total peptide counts, spectral counts (for C domain and full length), annotation, mass in kD, gene name, role, and localization of identified proteins are indicated. The full list of proteins identified is indicated in Appendix A (Supplemental Table S1).

Total Peptide Count	Spectral Counts	Total Peptide Count	Spectral Counts	Annotation	Mass	Gene	Role/Localization
C Domain	C Domain	Full Length	Full Length		(kD)		
31	**58**	43	**71**	**aminopeptidase N precursor [*Homo sapiens*]—CD13**	**109**	**ANPEP**	**Ectoproteinase/Membrane Raft**
8	**16**	28	**36**	**receptor-type tyrosine-protein phosphatase C isoform 2 precursor [*Homo sapiens*]—CD45**	**131**	**PTPRC**	**Phosphatase, signaling/Membrane**
4	**4**	21	**24**	**transferrin receptor protein 1 [*Homo sapiens*]—CD71**	**85**	**TFRC**	**Transporter/Membrane**
17	**21**	21	**27**	**4F2 cell-surface antigen heavy chain isoform c [*Homo sapiens*]—CD98**	**68**	**SLC3A2**	**Transporter/Membrane**
14	**15**	17	**23**	**leucyl-cystinyl aminopeptidase isoform 1 [*Homo sapiens*]**	**117**	**LNPEP**	**Ectoproteinase/Membrane Raft**
2	**2**	17	**20**	**carboxypeptidase D isoform 1 precursor [*Homo sapiens*]**	**153**	**CPD**	**Ectoproteinase/Membrane**
10	**23**	23	**31**	**integrin alpha-5 precursor [*Homo sapiens*]—CD51**	**114**	**ITGA5**	**Signaling/Membrane**
10	**16**	7	**11**	**integrin alpha-4 precursor [*Homo sapiens*]—CD49d**	**115**	**ITGA4**	**Signaling/Membrane**
1	**1**	9	**15**	**sodium bicarbonate cotransporter 3 isoform c [*Homo sapiens*]—Na^+^/HCO_3_—cotransporter**	**123**	**SLC4A7**	**Transporter/Membrane**
9	**16**	10	**16**	**integrin beta-1 isoform 1A precursor [*Homo sapiens*]—CD29**	**88**	**ITGB1**	**Signaling/Membrane**
3	**3**	10	**15**	**neutral amino acid transporter B(0) isoform 1 [*Homo sapiens*]**	**57**	**SLC1A5**	**Transporter/Membrane**
6	**9**	8	**13**	**basigin isoform 2 [*Homo sapiens*]—CD147**	**29**	**BSG**	**Signaling/Membrane**
1	**1**	8	**9**	**cytochrome b-245 heavy chain [*Homo sapiens*]**	**65**	**CYBB**	**Transporter/Membrane**
***0***	***0***	20	***31***	*receptor-type tyrosine-protein phosphatase alpha isoform 1 precursor [Homo sapiens]*	*91*	*PTPRA*	*Phosphatase, signaling/Membrane*
***0***	***0***	18	***22***	*sodium/potassium-transporting ATPase subunit alpha-1 isoform c [Homo sapiens] ATPase/Na^+^/K^+^*	*113*	*ATP1A1*	*Transporter/Membrane*
***0***	***0***	20	***24***	*hornerin [Homo sapiens]*	*282*	*HRNR*	*Unknown/Cytoplasmic Granule*
***0***	***0***	11	***11***	*V-type proton ATPase 116 kDa subunit a isoform 1 isoform c [Homo sapiens]—ATPase/H^+^*	*96*	*ATP6V0A1*	*Transporter/Lysosome*
***0***	***0***	9	***11***	*multidrug resistance-associated protein 4 isoform 1 [Homo sapiens]—ATP-binding cassette 4*	*149*	*ABCC4*	*Transporter/Membrane*
***0***	***0***	11	***11***	*multidrug resistance-associated protein 1 [Homo sapiens]—ATP-binding cassette 1*	*171*	*ABCC1*	*Transporter/Membrane*
***0***	***0***	10	***18***	*leukosialin precursor [Homo sapiens]—CD43*	*40*	*SPN*	*Signaling/Membrane*

## Data Availability

Peptide spectra and raw data have been uploaded on the PRIDE database (accession number PXD000664, https://www.ebi.ac.uk/pride/, accessed and deposited 19 December 2013, publicly available 9 February 2018. The raw data supporting the conclusions of this article will be made available by the authors on request, or have been presented in the manuscript and Supplementary Information.

## Supplementary Materials

The following supporting information can be downloaded at: https://www.mdpi.com/article/10.3390/biom15091243/s1, Table S1: Raw MS data.

## Abbreviations

The following abbreviations are used in this manuscript:

Benzyl-α GalNAc	Benzyl 2-acetamido-2-deoxy-α-D-galactopyranoside
BSA	bovine serum albumin
CD45I	CD45 Inhibitor
CHO	Chinese hamster ovary
CRD	carbohydrate recognition domain
DMSO	dimethyl sulfoxide
Gal-8	human Galectin-8
Gal-8C	C-terminal domain of Gal-8
Gal-8N	N-terminal domain of Gal-8
Gal-8CM	R233H mutant of Gal-8
Gal-8NM	R69H mutant of Gal-8
HL-60	Human promyelocytic leukemia cell
PBS	Phosphate buffer saline
PBS-TX	Phosphate buffer saline with 1.5% Triton X-100
PI	propidium iodide
polyLacNAc	poly-N-acetyllactosamine (-3Galβ1-4GlcNAcβ1-)_n_
PS	phosphatidylserine
PTP	Protein Tyrosine Phosphatase
Strep-Alexa	Streptavidin-Alexa Fluor labeled
Strep-HRP	Streptavidin-horseradish peroxidase
TBS	Tris buffer saline
TBST	Tris buffer saline with 0.1% Tween

## Appendix A

***Legend to Supplemental Table S1.* Tab Comparison Gal8-Gal8C**: Comparison of protein found in Gal-8C and Gal-8 fractions. Mass spectrometry data of lactose-eluted fractions with both Gal-8 and Gal-8C affinity chromatography. Identified proteins using both the full length and the C domain of Gal-8 are in bold, and proteins found only with full-length Gal-8 are italicized. Total peptide counts, spectral counts (for C domain and full length), annotation, mass in kDa, gene name, role, and localization of identified proteins are indicated in each column. **Tab Comparison_HILIC-Gal8**: Comparison of polyLacNAc containing glycoproteins identified by mass spectrometry from HL-60 cells by hydrophilic interaction chromatography (HILIC) [62], and Galectin-8 columns in this study. Proteins identified by Gal-8 columns (Raw-Gal8-Method), by HILIC (Raw-HILIC-Method), common proteins found in both methods (VLOOKUP-Common-Raw-Proteins_Gal8/HILIC), counts of common proteins (Common-Raw-Proteins_Gal8/HILIC), counts of total proteins (Total-Raw-Gal8-Proteins), and percent of proteins found in both methods (Percent_Raw-Gal8VSHILIC) are indicated in each column.

## References

[1] Liu F.T., Stowell S.R. (2023). The role of galectins in immunity and infection. Nat. Rev. Immunol..

[2] Zhang N., Liu Q., Wang D., Wang X., Pan Z., Han B., He G. (2025). Multifaceted roles of Galectins: From carbohydrate binding to targeted cancer therapy. Biomark. Res..

[3] Cummings R.D., Rabinovich G.A., Stowell S.R., Vasta G., Varki A., Cummings R.D., Esko J.D., Stanley P., Hart G.W., Aebi M., Mohnen D., Kinoshita T., Packer N.H., Prestegard J.H. (2022). Galectins. Essentials of Glycobiology.

[4] Stowell S.R., Arthur C.M., Dias-Baruffi M., Rodrigues L.C., Gourdine J.P., Heimburg-Molinaro J., Ju T., Molinaro R.J., Rivera-Marrero C., Xia B. (2010). Innate immune lectins kill bacteria expressing blood group antigen. Nat. Med..

[5] Barondes S.H., Cooper D.N., Gitt M.A., Leffler H. (1994). Galectins. Structure and function of a large family of animal lectins. J. Biol. Chem..

[6] Stowell S.R., Karmakar S., Arthur C.M., Ju T., Rodrigues L.C., Riul T.B., Dias-Baruffi M., Miner J., McEver R.P., Cummings R.D. (2009). Galectin-1 induces reversible phosphatidylserine exposure at the plasma membrane. Mol. Biol. Cell.

[7] Stowell S.R., Qian Y., Karmakar S., Koyama N.S., Dias-Baruffi M., Leffler H., McEver R.P., Cummings R.D. (2008). Differential roles of galectin-1 and galectin-3 in regulating leukocyte viability and cytokine secretion. J. Immunol..

[8] Stowell S.R., Arthur C.M., Slanina K.A., Horton J.R., Smith D.F., Cummings R.D. (2008). Dimeric Galectin-8 induces phosphatidylserine exposure in leukocytes through polylactosamine recognition by the C-terminal domain. J. Biol. Chem..

[9] Karmakar S., Stowell S.R., Cummings R.D., McEver R.P. (2008). Galectin-1 signaling in leukocytes requires expression of complex-type N-glycans. Glycobiology.

[10] Stowell S.R., Karmakar S., Stowell C.J., Dias-Baruffi M., McEver R.P., Cummings R.D. (2007). Human galectin-1, -2, and -4 induce surface exposure of phosphatidylserine in activated human neutrophils but not in activated T cells. Blood.

[11] Arthur C.M., Rodrigues L.C., Baruffi M.D., Sullivan H.C., Cummings R.D., Stowell S.R. (2015). Detection of phosphatidylserine exposure on leukocytes following treatment with human galectins. Methods Mol. Biol..

[12] Collins S.J. (1987). The HL-60 promyelocytic leukemia cell line: Proliferation, differentiation, and cellular oncogene expression. Blood.

[13] Nagata S., Suzuki J., Segawa K., Fujii T. (2016). Exposure of phosphatidylserine on the cell surface. Cell Death Differ..

[14] Rabinovich G.A., Toscano M.A. (2009). Turning ‘sweet’ on immunity: Galectin-glycan interactions in immune tolerance and inflammation. Nat. Rev. Immunol..

[15] Robinson B.S., Arthur C.M., Evavold B., Roback E., Kamili N.A., Stowell C.S., Vallecillo-Zúniga M.L., Van Ry P.M., Dias-Baruffi M., Cummings R.D. (2019). The Sweet-Side of Leukocytes: Galectins as Master Regulators of Neutrophil Function. Front. Immunol..

[16] Thijssen V.L., Hulsmans S., Griffioen A.W. (2008). The galectin profile of the endothelium: Altered expression and localization in activated and tumor endothelial cells. Am. J. Pathol..

[17] Souchak J., Mohammed N.B.B., Lau L.S., Dimitroff C.J. (2024). The role of galectins in mediating the adhesion of circulating cells to vascular endothelium. Front. Immunol..

[18] Karlsson M., Zhang C., Mear L., Zhong W., Digre A., Katona B., Sjostedt E., Butler L., Odeberg J., Dusart P. (2021). A single-cell type transcriptomics map of human tissues. Sci. Adv..

[19] Troncoso M.F., Elola M.T., Blidner A.G., Sarrias L., Espelt M.V., Rabinovich G.A. (2023). The universe of galectin-binding partners and their functions in health and disease. J. Biol. Chem..

[20] Bhakta S.B., Lundgren S.M., Sesti B.N., Flores B.A., Akdogan E., Collins S.R., Mercer F. (2024). Neutrophil-like cells derived from the HL-60 cell-line as a genetically-tractable model for neutrophil degranulation. PLoS ONE.

[21] Collins S.J., Gallo R.C., Gallagher R.E. (1977). Continuous growth and differentiation of human myeloid leukaemic cells in suspension culture. Nature.

[22] Malavez-Cajigas S.J., Marini-Martinez F.I., Lacourt-Ventura M., Rosario-Pacheco K.J., Ortiz-Perez N.M., Velazquez-Perez B., De Jesús-Rojas W., Chertow D.S., Strich J.R., Ramos-Benítez M.J. (2024). HL-60 cells as a valuable model to study LPS-induced neutrophil extracellular traps release. Heliyon.

[23] Thomas M.L. (1989). The leukocyte common antigen family. Annu. Rev. Immunol..

[24] Elliott J.I., Surprenant A., Marelli-Berg F.M., Cooper J.C., Cassady-Cain R.L., Wooding C., Linton K., Alexander D.R., Higgins C.F. (2005). Membrane phosphatidylserine distribution as a non-apoptotic signalling mechanism in lymphocytes. Nat. Cell Biol..

[25] Priglinger C.S., Szober C.M., Priglinger S.G., Merl J., Euler K.N., Kernt M., Gondi G., Behler J., Geerlof A., Kampik A. (2013). Galectin-3 induces clustering of CD147 and integrin-beta1 transmembrane glycoprotein receptors on the RPE cell surface. PLoS ONE.

[26] Blenda A.V., Kamili N.A., Wu S.-C., Abel W.F., Ayona D., Gerner-Smidt C., Ho A.D., Benian G.M., Cummings R.D., Arthur C.M. (2022). Galectin-9 recognizes and exhibits antimicrobial activity toward microbes expressing blood group–like antigens. J. Biol. Chem..

[27] Blum H., Beier H., Gross H.J. (1987). Improved silver staining of plant proteins, RNA and DNA in polyacrylamide gels. Electrophoresis.

[28] Seyfried N.T., Gozal Y.M., Donovan L.E., Herskowitz J.H., Dammer E.B., Xia Q., Ku L., Chang J., Duong D.M., Rees H.D. (2012). Quantitative analysis of the detergent-insoluble brain proteome in frontotemporal lobar degeneration using SILAC internal standards. J. Proteome Res..

[29] Elias J.E., Gygi S.P. (2007). Target-decoy search strategy for increased confidence in large-scale protein identifications by mass spectrometry. Nat. Methods.

[30] Barsnes H., Vizcaino J.A., Eidhammer I., Martens L. (2009). PRIDE Converter: Making proteomics data-sharing easy. Nat. Biotechnol..

[31] Stanton T., Boxall S., Hirai K., Dawes R., Tonks S., Yasui T., Kanaoka Y., Yuldasheva N., Ishiko O., Bodmer W. (2003). A high-frequency polymorphism in exon 6 of the CD45 tyrosine phosphatase gene (PTPRC) resulting in altered isoform expression. Proc. Natl. Acad. Sci. USA.

[32] Lynch K.W., Weiss A. (2000). A model system for activation-induced alternative splicing of CD45 pre-mRNA in T cells implicates protein kinase C and Ras. Mol. Cell Biol..

[33] Laemmli U.K. (1970). Cleavage of structural proteins during the assembly of the head of bacteriophage T4. Nature.

[34] Nishi N., Shoji H., Seki M., Itoh A., Miyanaka H., Yuube K., Hirashima M., Nakamura T. (2003). Galectin-8 modulates neutrophil function via interaction with integrin αM. Glycobiology.

[35] Si Y., Zhu J., Sayed H., Mayo K.H., Zhou Y., Tai G., Su J. (2025). CD98hc, a novel of galectin-8 receptor, binds to galectin-8 in an N-glycosylation-dependent manner. Acta Biochim. Biophys. Sin..

[36] Fujiwara S., Shinkai H., Deutzmann R., Paulsson M., Timpl R. (1988). Structure and distribution of N-linked oligosaccharide chains on various domains of mouse tumour laminin. Biochem. J..

[37] Knibbs R.N., Perini F., Goldstein I.J. (1989). Structure of the major concanavalin A reactive oligosaccharides of the extracellular matrix component laminin. Biochemistry.

[38] Stanley P., Sundaram S., Sallustio S. (1991). A subclass of cell surface carbohydrates revealed by a CHO mutant with two glycosylation mutations. Glycobiology.

[39] Osset M., Piñol M., Fallon M.J., de Llorens R., Cuchillo C.M. (1989). Interference of the carbohydrate moiety in coomassie brilliant blue R-250 protein staining. Electrophoresis.

[40] Takaishi M., Makino T., Morohashi M., Huh N.-H. (2005). Identification of Human Hornerin and Its Expression in Regenerating and Psoriatic Skin. J. Biol. Chem..

[41] Okamoto T., Hattori M., Katsube Y., Ota J., Asanuma K., Usuda H., Wada K., Suzuki K., Nikai T. (2025). Hornerin expressed on endothelial cells via interacting with thrombomodulin modulates vascular inflammation and angiogenesis. Biochim. Biophys. Acta (BBA) Mol. Cell Res..

[42] Gutknecht M.F., Seaman M.E., Ning B., Cornejo D.A., Mugler E., Antkowiak P.F., Moskaluk C.A., Hu S., Epstein F.H., Kelly K.A. (2017). Identification of the S100 fused-type protein hornerin as a regulator of tumor vascularity. Nat. Commun..

[43] Fleming J.M., Ginsburg E., Oliver S.D., Goldsmith P., Vonderhaar B.K. (2012). Hornerin, an S100 family protein, is functional in breast cells and aberrantly expressed in breast cancer. BMC Cancer.

[44] Cotter K., Stransky L., McGuire C., Forgac M. (2015). Recent Insights into the Structure, Regulation, and Function of the V-ATPases. Trends Biochem. Sci..

[45] Eaton A.F., Merkulova M., Brown D. (2021). The H^+^-ATPase (V-ATPase): From proton pump to signaling complex in health and disease. Am. J. Physiol. Cell Physiol..

[46] Taetle R., Ostergaard H., Smedsrud M., Trowbridge I. (1991). Regulation of CD45 expression in human leukemia cells. Leukemia.

[47] Hermiston M.L., Xu Z., Weiss A. (2003). CD45: A Critical Regulator of Signaling Thresholds in Immune Cells. Annu. Rev. Immunol..

[48] Zick Y. (2022). Galectin-8, cytokines, and the storm. Biochem. Soc. Trans..

[49] Anderson N.G., Maller J.L., Tonks N.K., Sturgill T.W. (1990). Requirement for integration of signals from two distinct phosphorylation pathways for activation of MAP kinase. Nature.

[50] Tribulatti M.V., Cattaneo V., Hellman U., Mucci J., Campetella O. (2009). Galectin-8 provides costimulatory and proliferative signals to T lymphocytes. J. Leukoc. Biol..

[51] Romaniuk M.A., Tribulatti M.V., Cattaneo V., Lapponi M.J., Molinas F.C., Campetella O., Schattner M. (2010). Human platelets express and are activated by galectin-8. Biochem. J..

[52] Alge-Priglinger C.S., Andre S., Schoeffl H., Kampik A., Strauss R.W., Kernt M., Gabius H.J., Priglinger S.G. (2011). Negative regulation of RPE cell attachment by carbohydrate-dependent cell surface binding of galectin-3 and inhibition of the ERK-MAPK pathway. Biochimie.

[53] Elbein A.D., Tropea J.E., Mitchell M., Kaushal G.P. (1990). Kifunensine, a potent inhibitor of the glycoprotein processing mannosidase I. J. Biol. Chem..

[54] Huang J., Byrd J.C., Yoon W.H., Kim Y.S. (1992). Effect of benzyl-alpha-GalNAc, an inhibitor of mucin glycosylation, on cancer-associated antigens in human colon cancer cells. Oncol. Res..

[55] Walcheck B., Leppanen A., Cummings R.D., Knibbs R.N., Stoolman L.M., Alexander S.R., Mattila P.E., McEver R.P. (2002). The monoclonal antibody CHO-131 binds to a core 2 O-glycan terminated with sialyl-Lewis x, which is a functional glycan ligand for P-selectin. Blood.

[56] van Heyningen S. (1977). Cholera toxin. Biol. Rev. Camb. Philos. Soc..

[57] Baenziger J.U., Fiete D. (1979). Structural determinants of concanavalin A specificity for oligosaccharides. J. Biol. Chem..

[58] Cummings R.D., Kornfeld S. (1982). Characterization of the structural determinants required for the high affinity interaction of asparagine-linked oligosaccharides with immobilized Phaseolus vulgaris leukoagglutinating and erythroagglutinating lectins. J. Biol. Chem..

[59] Plummer T.H., Elder J.H., Alexander S., Phelan A.W., Tarentino A.L. (1984). Demonstration of peptide:N-glycosidase F activity in endo-beta-N-acetylglucosaminidase F preparations. J. Biol. Chem..

[60] Tang W., Chang S.B., Hemler M.E. (2004). Links between CD147 function, glycosylation, and caveolin-1. Mol. Biol. Cell.

[61] Mitsui Y., Yamada K., Hara S., Kinoshita M., Hayakawa T., Kakehi K. (2012). Comparative studies on glycoproteins expressing polylactosamine-type N-glycans in cancer cells. J. Pharm. Biomed. Anal..

[62] Togayachi A., Tomioka A., Fujita M., Sukegawa M., Noro E., Takakura D., Miyazaki M., Shikanai T., Narimatsu H., Kaji H. (2018). Identification of Poly-N-Acetyllactosamine-Carrying Glycoproteins from HL-60 Human Promyelocytic Leukemia Cells Using a Site-Specific Glycome Analysis Method, Glyco-RIDGE. J. Am. Soc. Mass. Spectrom..

[63] Piedfer M., Dauzonne D., Tang R., N’Guyen J., Billard C., Bauvois B. (2011). Aminopeptidase-N/CD13 is a potential proapoptotic target in human myeloid tumor cells. FASEB J..

[64] Mina-Osorio P. (2008). The moonlighting enzyme CD13: Old and new functions to target. Trends Mol. Med..

[65] Sato T., Furukawa K., Autero M., Gahmberg C.G., Kobata A. (1993). Structural study of the sugar chains of human leukocyte common antigen CD45. Biochemistry.

[66] Earl L.A., Bi S., Baum L.G. (2010). N- and O-glycans modulate galectin-1 binding, CD45 signaling, and T cell death. J. Biol. Chem..

[67] Ferguson B.V., Ostergaard H.L. (2010). CD45 regulates thymocyte survival during development in fetal thymic organ culture. Immunobiology.

[68] Gao H., Henderson A., Flynn D.C., Landreth K.S., Ericson S.G. (2000). Effects of the protein tyrosine phosphatase CD45 on FcgammaRIIa signaling and neutrophil function. Exp. Hematol..

[69] Panchal R.G., Ulrich R.L., Bradfute S.B., Lane D., Ruthel G., Kenny T.A., Iversen P.L., Anderson A.O., Gussio R., Raschke W.C. (2009). Reduced expression of CD45 protein-tyrosine phosphatase provides protection against anthrax pathogenesis. J. Biol. Chem..

[70] Chen I.J., Chen H.L., Demetriou M. (2007). Lateral compartmentalization of T cell receptor versus CD45 by galectin-N-glycan binding and microfilaments coordinate basal and activation signaling. J. Biol. Chem..

[71] Haston W.S., Maggs A.F. (1990). Evidence for membrane differentiation in polarised leucocytes: The distribution of surface antigens analysed with Ig-gold labelling. J. Cell Sci..

[72] Seveau S., Eddy R.J., Maxfield F.R., Pierini L.M. (2001). Cytoskeleton-dependent membrane domain segregation during neutrophil polarization. Mol. Biol. Cell.

[73] Cattaneo V., Tribulatti M.V., Campetella O. (2011). Galectin-8 tandem-repeat structure is essential for T-cell proliferation but not for co-stimulation. Biochem. J..

[74] Pulido R., Lacal P., Mollinedo F., Sanchez-Madrid F. (1989). Biochemical and antigenic characterization of CD45 polypeptides expressed on plasma membrane and internal granules of human neutrophils. FEBS Lett..

[75] Kirk P., Wilson M.C., Heddle C., Brown M.H., Barclay A.N., Halestrap A.P. (2000). CD147 is tightly associated with lactate transporters MCT1 and MCT4 and facilitates their cell surface expression. EMBO J..

[76] Wilson M.C., Meredith D., Fox J.E., Manoharan C., Davies A.J., Halestrap A.P. (2005). Basigin (CD147) is the target for organomercurial inhibition of monocarboxylate transporter isoforms 1 and 4: The ancillary protein for the insensitive MCT2 is EMBIGIN (gp70). J. Biol. Chem..

[77] Feral C.C., Nishiya N., Fenczik C.A., Stuhlmann H., Slepak M., Ginsberg M.H. (2005). CD98hc (SLC3A2) mediates integrin signaling. Proc. Natl. Acad. Sci. USA.

[78] Kasinrerk W., Fiebiger E., Stefanova I., Baumruker T., Knapp W., Stockinger H. (1992). Human leukocyte activation antigen M6, a member of the Ig superfamily, is the species homologue of rat OX-47, mouse basigin, and chicken HT7 molecule. J. Immunol..

[79] Kato N., Yuzawa Y., Kosugi T., Hobo A., Sato W., Miwa Y., Sakamoto K., Matsuo S., Kadomatsu K. (2009). The E-selectin ligand basigin/CD147 is responsible for neutrophil recruitment in renal ischemia/reperfusion. J. Am. Soc. Nephrol..

[80] Fukuda M., Matsumura G. (1975). Endo-beta-galactosidase of Escherichia freundii. Hydrolysis of pig colonic mucin and milk oligosaccharides by endoglycosidic action. Biochem. Biophys. Res. Commun..

[81] Rillahan C.D., Antonopoulos A., Lefort C.T., Sonon R., Azadi P., Ley K., Dell A., Haslam S.M., Paulson J.C. (2012). Global metabolic inhibitors of sialyl- and fucosyltransferases remodel the glycome. Nat. Chem. Biol..

[82] Lee N., Wang W.C., Fukuda M. (1990). Granulocytic differentiation of HL-60 cells is associated with increase of poly-N-acetyllactosamine in Asn-linked oligosaccharides attached to human lysosomal membrane glycoproteins. J. Biol. Chem..

[83] Mizoguchi A., Takasaki S., Maeda S., Kobata A. (1984). Changes in asparagine-linked sugar chains of human promyelocytic leukemic cells (HL-60) during monocytoid differentiation and myeloid differentiation. Appearance of high mannose-type oligosaccharides in neutral fraction. J. Biol. Chem..

[84] Wilkins P.P., McEver R.P., Cummings R.D. (1996). Structures of the O-glycans on P-selectin glycoprotein ligand-1 from HL-60 cells. J. Biol. Chem..

[85] Fossum S., Mallett S., Barclay A.N. (1991). The MRC OX-47 antigen is a member of the immunoglobulin superfamily with an unusual transmembrane sequence. Eur. J. Immunol..

[86] Spring F.A., Holmes C.H., Simpson K.L., Mawby W.J., Mattes M.J., Okubo Y., Parsons S.F. (1997). The Oka blood group antigen is a marker for the M6 leukocyte activation antigen, the human homolog of OX-47 antigen, basigin and neurothelin, an immunoglobulin superfamily molecule that is widely expressed in human cells and tissues. Eur. J. Immunol..

[87] Biswas C., Zhang Y., DeCastro R., Guo H., Nakamura T., Kataoka H., Nabeshima K. (1995). The human tumor cell-derived collagenase stimulatory factor (renamed EMMPRIN) is a member of the immunoglobulin superfamily. Cancer Res..

[88] Yurchenko V., O’Connor M., Dai W.W., Guo H., Toole B., Sherry B., Bukrinsky M. (2001). CD147 is a signaling receptor for cyclophilin B. Biochem. Biophys. Res. Commun..

[89] Pushkarsky T., Zybarth G., Dubrovsky L., Yurchenko V., Tang H., Guo H., Toole B., Sherry B., Bukrinsky M. (2001). CD147 facilitates HIV-1 infection by interacting with virus-associated cyclophilin A. Proc. Natl. Acad. Sci. USA.

[90] Berditchevski F., Chang S., Bodorova J., Hemler M.E. (1997). Generation of monoclonal antibodies to integrin-associated proteins. Evidence that alpha3beta1 complexes with EMMPRIN/basigin/OX47/M6. J. Biol. Chem..

[91] Sakuragi T., Kanai R., Tsutsumi A., Narita H., Onishi E., Nishino K., Miyazaki T., Baba T., Kosako H., Nakagawa A. (2021). The tertiary structure of the human Xkr8-Basigin complex that scrambles phospholipids at plasma membranes. Nat. Struct. Mol. Biol..

[92] Suzuki J., Denning D.P., Imanishi E., Horvitz H.R., Nagata S. (2013). Xk-related protein 8 and CED-8 promote phosphatidylserine exposure in apoptotic cells. Science.

[93] Suzuki J., Imanishi E., Nagata S. (2016). Xkr8 phospholipid scrambling complex in apoptotic phosphatidylserine exposure. Proc. Natl. Acad. Sci. USA.

[94] Levy Y., Arbel-Goren R., Hadari Y.R., Eshhar S., Ronen D., Elhanany E., Geiger B., Zick Y. (2001). Galectin-8 functions as a matricellular modulator of cell adhesion. J. Biol. Chem..

[95] Hadari Y.R., Arbel-Goren R., Levy Y., Amsterdam A., Alon R., Zakut R., Zick Y. (2000). Galectin-8 binding to integrins inhibits cell adhesion and induces apoptosis. J. Cell Sci..

[96] McCall M.N., Uppal K., Jaffee H.A., Zilliox M.J., Irizarry R.A. (2011). The Gene Expression Barcode: Leveraging public data repositories to begin cataloging the human and murine transcriptomes. Nucleic Acids Res..

[97] Marino G., Kroemer G. (2013). Mechanisms of apoptotic phosphatidylserine exposure. Cell Res..

[98] Karmakar S., Cummings R.D., McEver R.P. (2005). Contributions of Ca2+ to galectin-1-induced exposure of phosphatidylserine on activated neutrophils. J. Biol. Chem..

